# Relationships of morphological and phototextural attributes of presumptive ovine zygotes and early embryos to their developmental competence *in vitro*: a preliminary assessment using time-lapse imaging

**DOI:** 10.1590/1984-3143-AR2022-0009

**Published:** 2022-04-08

**Authors:** Karolina Fryc, Agnieszka Nowak, Barbara Kij-Mitka, Joanna Kochan, Maciej Murawski, Samantha Pena, Pawel Mieczyslaw Bartlewski

**Affiliations:** 1 Department of Animal Nutrition, Biotechnology and Fisheries, University of Agriculture in Kraków, Cracow, Poland; 2 Department of Animal Reproduction, Anatomy and Genomics, University of Agriculture in Kraków, Cracow, Poland; 3 Department of Biomedical Sciences, Ontario Veterinary College, University of Guelph, Guelph, ON, Canada

**Keywords:** sheep, *in vitro* embryo production, time-lapse imaging, morphology, phototexture

## Abstract

The assessment of morphology and digital image opacity may provide valuable information on the present embryo quality. Time-lapse imaging has been employed in research to establish a means of monitoring the dynamic nature of preimplantation embryo development. The aim of present study was to use time-lapse imaging for assessing various prospective morphometric and phototextural markers of the developmental potential of *in vitro*-derived ovine embryos. Oocytes were obtained by scarification of ovaries from nine Polish Longwool ewes. After *in vitro* maturation (IVM) and fertilization (IVF) of oocytes with fresh ram semen, the development of embryos to the blastocyst stage was monitored and evaluated using Primo Vision time-lapse imaging technology. Commercially available Image-Pro^®^ Plus software was used to measure zona pellucida thickness, embryo diameter, total area of the perivitelline space, cellular grey-scale pixel intensity and cellular pixel heterogeneity. Statistical assessment of all attributes was done at various time points during embryo development (i.e., presumptive zygote stage: t(0); first cleavage detected at t(2) or t(3); and second cleavage detected at t(4) or t(6)). Out of thirty-seven zygotes analyzed in this study, five did not divide, 26 arrested before and six developed to the blastocyst stage. Our present results indicate that most parameters analyzed did not differ among embryos varying in their developmental fate except for the perivitelline space area that was greater (P<0.05) for non-dividing zygotes than future blastocysts at the presumptive zygote stage (4040±1850 vs. 857±262 µm^2^, respectively; means±SEM). Consequently, the measurement of perivitelline space at t(0) can potentially be used to prognosticate developmental potential of *in vitro*-produced ovine embryos albeit further confirmational studies are needed.

## Introduction

Time-lapse imaging of *in vitro*-derived embryos is a technology used to collect and analyze morphological data obtained at frequent intervals from digital images during the preimplantation period of embryonic development, without disruption of the embryo culture environment (Mandawala et al., 2016). This method has been implemented due mainly to its ability to encompass all cleavage events and analyze the timing of successive progressions throughout early embryogenesis (Gardner and Balaban, 2016). For example, using time-lapse imaging, it was observed that sheep embryos developing to the blastocyst stage underwent cleavage divisions and attained the morula stage earlier than arresting embryos (Fryc et al., 2021). Time-lapse imaging offers a fully automated system that removes subjectivity and minimizes several adverse extrinsic factors associated with intermittent researchers’ observations (Feyeux et al., 2020). Hence, time-lapse imaging is a plausible alternative to and replacement for the standard light microscopy in assessing morphological and functional markers of embryo quality.

As the embryos are frequently produced *in vitro* with the intention of ensuing pregnancy and live births, selection of the most viable embryo for transfer to surrogate mothers or synchronized recipients is of paramount importance (Nasiri and Eftekhari-Yazdi, 2015; Gardner and Balaban, 2016; Zaninovic et al., 2017). Up to date, embryo selection has relied primarily on morphological criteria evaluated with alphanumeric scoring systems, such as Gardner and Schoolcraft’s grading system (Gardner and Schoolcraft, 1999). Unfortunately, this technique is associated with potential human error, and is not completely free of biases and confounding influences.

In the search for alternative, non-invasive methods of embryo assessment, our study employed the phototexture analysis of ovine embryos. The term phototexture refers to the visual quality of the surface of an object, revealed through variances in contrast and tone (Vanduzer et al., 2014). In the past, phototexture could only be examined visually, but presently pixel intensity and uniformity of digital images can be assessed using computerized image analysis. Visual assessment is prone to inconsistencies and heavily dependent on experience of the assessors, and hence may not always be adequate for diagnostic purposes, whereas computer-aided grey-scale image analysis provides a quantitative approach that reduces such inconsistencies (Singh et al., 1998; VerMilyea et al., 2020; Zaninovic and Rosenwaks, 2020). Several factors including chemical composition (e.g., lipid content) and intracellular architecture of blastomeres could influence the appearance of the mammalian embryo (Van Soom et al., 2003). Such factors can vary tremendously among individual embryos and impinge on their viability (Van Soom et al., 2003), but there has been no systematic study of embryonic cell phototexture in relation to their *in vitro* developmental potential.

The aim of this study was to assess select morphometric and phototextural parameters of presumptive zygotes and pre-morula stages to determine the ability of *in vitro-* produced ovine embryos to attain the blastocyst stage. The characteristics under investigation included zona pellucida thickness, embryo diameter, perivitelline space area, and cellular pixel intensity and heterogeneity. The use of time-lapse imaging permitted uninterrupted analysis of embryos at specific developmental stages. Based on the comparisons between *in vivo*- and *in vitro*-derived bovine embryos (Van Soom et al., 2003), we anticipated that a thinner zona pellucida, larger zygote/embryo, homogeneous blastomeres with high pixel intensity, and larger perivitelline space would be correlated with an uninterrupted development of ovine embryos to the blastocyst stage.

## Methods

### *Oocyte collection and* in vitro *maturation*

**Figure 1:**
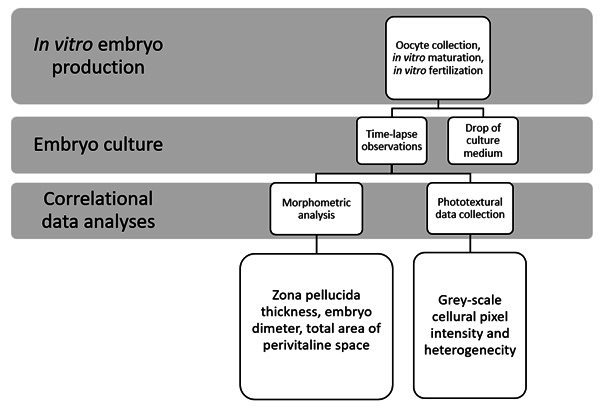
An overview of sheep embryo culture procedures as well as morphological and phototexture analyses.

The present experimental procedures are outlined in Figure 1. This study was performed following the international guidelines on animal care and use for research, and since it utilized the slaughterhouse material, the present project did not require ethical approval. Nine Longwool breed ewes from 1.5 to 3 years of age and in good body condition score were slaughtered during the breeding season (October-March) in a local abattoir near Cracow, Poland. Ovaries were kept in phosphate-buffered saline (PBS) buffer solution pre-warmed to 30-35^o^C during transportation to the laboratory, for a maximum period of 3 h prior to oocyte collection. All chemicals were purchased from the Sigma-Aldrich (Merck KGaA, Darmstadt, Germany) unless otherwise stated. Cumulus oocyte complexes (COCs) were retrieved from scarified ovaries that had been pre-washed in fresh PBS at 37^o^C. Oocytes with a minimum of four layers of surrounding granulosa cells were selected for *in vitro* maturation (IVM) performed for 24 h in media containing TCM 199 with Earle’s Salt, 10% FBS, 5 µg/ml of ovine luteinizing hormone (0.1 IU/ml), 5 µg/ml of ovine follicle-stimulating hormone (0.1 IU/ml), and 1 μg/ml AAS (antibiotic/antimycotic solution containing penicillin and streptomycin) at 5% CO_2_ and 100% humidity at 38^o^C.

### In vitro *fertilization and embryo culture*

A mixture containing 10 µl of fresh ram semen collected from the three rams of Polska Owca Pogórza breed with previously confirmed fertility and 1 ml of Sperm Air medium (Gynemed, Lensahn, Germany) was centrifuged at 2500 rpm for 7 min. The resulting supernatant was combined with another 1 ml of Fresh Sperm Air medium. Semen was then incubated at 38^o^C for 30-40 min under atmospheric conditions of 5% CO_2_ to allow sperm capacitation, and capacitated spermatozoa were then subjected to the swim-up method (Jameel, 2008). COCs were co-incubated with 1 x 10^6^ sperm/ml for 19 h in Cult medium (Gynemed, Lensahn, Germany), and atmosphere of 5% CO_2_ in humidified air at 38^o^C. Following *in vitro* fertilization (IVF), presumptive zygotes were randomly transferred to 50-µl drops of Cult medium (Gynemed, Lensahn, Germany) in 16-well dishes under mineral oil (Gynemed, Lensahn, Germany) according to the procedure described by Kochan et al. (2022). Presumptive zygotes and developing embryos were left undisturbed for 8 days, except briefly every 48 h to change 30 µl of culture media.

### Time-lapse imaging, morphometric and phototextural data collection

**Figure 2:**
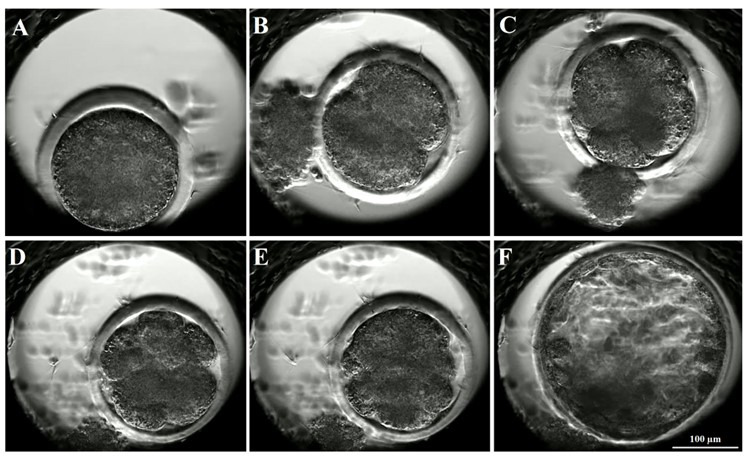
Digital images of ovine embryonic development from presumptive zygote to blastocyst captured by time-lapse imaging system. (A) presumptive zygote, t(0); (B) two-cell embryo, t(2); (C) four-cell embryo, t(4); (D) eight-cell embryo, t(8); (E) morula, t(M); and (F) blastocyst.

Primo Vision time-lapse system (EAVO+; Vitrolife, Göteburg, Sweden) was used to monitor embryonic development by taking digital images every 10 min for 8 days post-fertilization. Digital images of ovine embryos (Figure 2) at critical developmental time points (i.e., presumptive zygotes, t(0); first cleavage, t(2) or t(3); second cleavage, t(4) or t(6); third cleavage, t(7) or t(8); and morula stage, t(M)) were saved.

**Figure 3:**
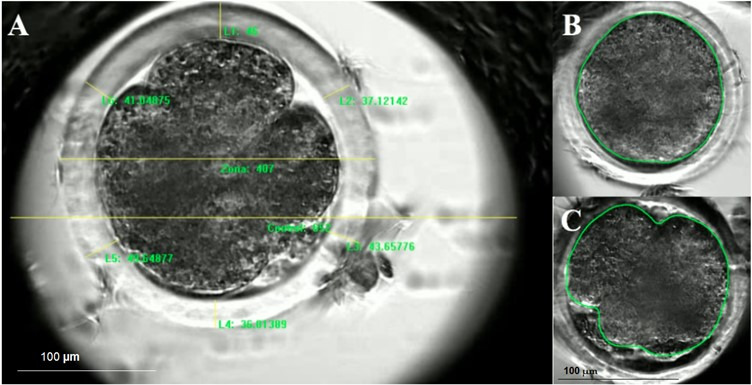
Digital images of ovine embryos assessed using Image-Pro^®^ Plus analytical software. (A) Raw morphometric data, computed in pixels, of a four-cell embryo. Six randomly selected points on the zona pellucida, one measurement of the diameter of the embryo, and one measurement of the diameter of the field of view; (B) and (C) polygonal tool used for phototexture analyses of a presumptive zygote and a four-cell embryo, respectively.


The following morphometric attributes of presumptive zygotes and dividing embryos were measured using Image-Pro^®^Plus ver. 7.0 analytical software (Media Cybernetics Inc., Rockville, MD, USA): thickness of the zona pellucida, embryo diameter, and total area of the perivitelline space (Figure 3). The diameter of the well was computed as a control or reference point to calculate embryo dimensions. Pixel values were converted to SI units (i.e., μm) based on the magnification of the time-lapse imager (i.e., 100x) and the diameter of the exposure area (i.e., 2.5 mm). Each measurement in pixels (z) was divided by the control measurement (c), and then multiplied by the magnification and exposure area to yield a value in SI units (y). The following equation was used:

$$Y=\left[\;\frac{z}{c}\;\right]\;\times \;100\;\times \;2.5$$ (1)

Cellular pixel intensity (CPI; Figure 3) values were normalized to account for variations in the limits of the scale from 0 to 255 (representing absolute black and absolute white pixels, respectively), using the following equation (Singh et al., 1998):


$$NCPI=\left[CPI-xmin\right]\times \left[\frac{255}{xmax-xmin}\right]\left[CPI-xmin\right]\times \left[\frac{255}{xmax-xmin}\right]$$ (2)

with the lowest pixel value (*xmin*) and the highest pixel value (*xmax*) recorded in each grey-scale digital image; (NCPI), normalized cell pixel intensity.

### Statistical analyses

One-way analysis of variance (ANOVA) was done using (SigmaPlot^®^11.0; Systat Software Inc., San Jose, CA, USA) for all variables/embryonic fates at the presumptive zygote stage, t(0), and the differences between future arresting and non-arresting embryos after first cleavage, at t(2) or t(3), and second cleavage, t(4) or t(6), were analyzed by Student *t*-test. Pairwise comparisons by the Holm-Sidak method were conducted if the ANOVA indicated significance (P<0.05) for between-group variances. All results are expressed as mean±standard error of the mean (SEM) unless otherwise indicated.

## Results

### *Assessment of* in vitro*-derived ovine embryos*

Eighty-eight oocytes of excellent and good quality were obtained from 9 slaughtered ewes in 3 independent replicates (Table 1). Thirty-seven presumptive zygotes were randomly selected for time-lapse observations. The remaining presumptive zygotes (n=51) were cultured in drops of Cult medium under mineral oil out of which 43 underwent the first cleavage division (cleavage rate of 84.3%) and 13 developed to blastocyst stage (blastocyst formation rate of 30.2%). Out of 37 embryos used for time-lapse imaging, five did not divide (cleavage rate of 86.5%), nine developed to the 2-cell stage, two to the 3-cell stage, eight to the 4-cell stage, one to the 5-cell stage, two to the 6-cell stage, four to the 7-cell stage, and six to the blastocyst stage (blastulation rate of 21.9%). No arrest occurred from the 8-cell stage to the blastocyst of the ovine embryos studied (Table 1).

**Table 1:** Numbers of ovine embryos monitored with time-lapse imaging technology and organized by study replicates/donor ewes and developmental fate.

**Replicate**	**Number of ewes**	**Total no. of embryos**	**Developmental fate of embryos**		
			**Non-dividing**	**Cleavage-stage arresting**	**Blastocysts**
1	3	13	3	9	1
2	3	10	2	5	3
3	3	14	0	12	2
Total	9	37	5	26	6

### Morphometric and phototextural analyses

Total perivitelline space area that was greater (P<0.05) for non-dividing zygotes compared with that of future blastocysts at t(0) (4040±1850 vs. 857±262 µm^2^, respectively; Table 2). At any stage (t(0), t(2) or t(3) and t(4) or t(6)), none of the other attributes analyzed in this experiment varied between the presumptive ovine zygotes or early embryos of different developmental success (P>0.05).

**Table 2:** Summary of morphometric and phototextural attributes of the developing ovine embryos evaluated at three different time points or developmental stages: presumptive zygotes: t(0); first cleavage: t(2) or t(3); and second cleavage: t(4) or t(6); *P<0.05.

**Embryo categories/Variables**	**Zona pellucida thickness (µm)**	**Embryo diameter (µm)**	**Perivitelline space area (µm^2^)**	**Cellular pixel intensity (0-255)**	**Cellular pixel heterogeneity (0-255)**
	**Presumptive zygotes**				
Non-dividing (n=5)	14.3±1.0	161.8±3.9	4040±1850*	68.6±2.7	30.7±1.4
Blastocysts (n=6)	13.6±0.6	152.9±2.9	857±262*	73.7±2.5	31.5±1.1
All arresting (n=26)	13.5±0.3	154.4±1.4	2139±356	76.7±2.5	32.0±1.1
			First cleavage		
Blastocyst (n=6)	12.6±1.1	152.9±2.5	1443±256	74.5±2.5	30.3±1.6
All arresting (n=26)	13.5±0.4	156.8±2.0	2504±299	75.1±2.5	32.8±1.7
			Second cleavage		
Blastocyst (n=6)	12.8±0.7	150.9±3.1	1731±206	72.5±3.6	30.0±1.4
Arresting at t(4-7) (n=15)	13.4±0.5	152.0±2.0	2096±449	72.1±5.0	31.8±1.5

## Discussion

In this study, we confirmed the utility of using time-lapse imaging technology to monitor the development of ovine embryos from fertilization to the blastocyst stage. Frequent acquisition of images allowed us to create data base for further exploration of the factors that can influence ovine embryogenesis *in vitro*. Previous literature has demonstrated that most mammalian embryos will have arrested by the morula stage because of asymmetry. Following this critical time point, embryos are considerably more tolerant to abnormal cleavage events (Cruz et al., 2012; Lagalla et al., 2017; Kij-Mitka et al., 2019, 2020). Notably, in the current study, the development past the 7-cell stage consistently led to ovine blastocyst formation. This agrees with a study by Burruel et al. (2014) who demonstrated that most human embryos arrested by the 8-cell stage of development. Consequently, the current study analyzed morphometric and phototextural attributes up to the second cleavage stage, t(4) or t(6), and measurements were not analyzed following the later stages due to the fact that no morphological indicators of blastocyst forming ability/embryonic arrest would be detected at or after t(7).

In conjunction with time-lapse imaging, Image-Pro^®^ Plus software served as a useful tool for quantitative, morphological assessment of various attributes captured in the digital images. Overall, the interpretation of the present findings suggests that a majority of morphometric and phototextural attributes of presumptive zygotes and early embryos are poor predictors of the developmental potential of ovine embryos. Zona pellucida thickness, diameter of the embryo, and cellular phototexture did not vary significantly between embryos with different developmental fates. However, a larger perivitelline space was found in non-dividing ovine embryos compared to future blastocysts assessed at the presumptive zygote stage; the difference was significant despite the low number of presumptive zygotes assessed and high variability in perivitelline space observed in non-dividing embryos at t(0).

The exact mechanism and implications of perivitelline space organization for embryo development remain unknown. In earlier studies using porcine and mouse oocytes, the perivitelline space was reported to be significantly larger in ovulated oocytes than in oocytes cultured *in vitro* (Wang et al., 1998; Ueno and Niimura, 2008). A study of rabbit and hamster oocytes suggested that the smaller size of the perivitelline space was closely related to the incidence of polyspermy (Yoshida and Niimura, 2010). Alternatively, in a study of human oocytes, a large perivitelline space was found to be one of the most significant factors associated with lower fertilization rates (Rienzi et al., 2005). Culture media composition may also influence the size of the perivitelline space. Glucuronic acid and N-acetyl-D-glucosamine supplementation during *in vitro* maturation of porcine oocytes has been shown to increase the size of the perivitelline space, which was associated with a decrease in the percentage of polyspermic zygotes (Current and Whitaker, 2020).

Variations in the amount and distribution of lipid content in oocytes across various mammalian species (i.e., sheep, pigs, cows, humans, and mice) have been indicators of their developmental competence *in vitro* (Dunning et al., 2014; Aizawa et al., 2019). Quantifying lipid content remains an important indicator of suboptimal culture conditions and hence a predictor of embryo fate and developmental potential. As previously demonstrated by Jeong et al. (2009), oocyte color, as a measure of lipid content, can be predictive of embryo developmental potential. Kochan et al. (2021a, b) have shown that oocytes with light cytoplasm were significantly smaller than oocytes with dark cytoplasm in adult and prepubertal cats. Low light permeation of oocytes reflects their high lipid content (Jeong et al., 2009). Therefore, we stipulated that measurements of phototextural attributes of embryonic cells would reflect embryonic lipid content. Unfortunately, our observations did not support the null hypothesis that photometric characteristics of presumptive ovine zygotes and/or developing embryos would differ among embryos varying in their developmental potential.

## Conclusion

In summary, the current pilot study has provided precedence for morphological assessment of ovine presumptive zygotes and early embryos as prospective biomarkers of their ability to reach the blastocyst stage. Specifically, our present results posit that automated measurement of perivitelline space area immediately post-fertilization may be a useful method of selecting future viable ovine blastocysts. Non-invasiveness and simplicity of such an approach are extremely appealing and cost effective both in clinical practice and agri-business.
